# Is outdoor work associated with elevated rates of cerebrovascular disease mortality? A cohort study based on iron-ore mining

**DOI:** 10.1186/s12995-016-0131-8

**Published:** 2016-08-26

**Authors:** Ove Björ, Håkan Jonsson, Lena Damber, Lage Burström, Tohr Nilsson

**Affiliations:** 1 Department of Radiation Sciences (Oncology), Umeå University, SE-901 85 Umeå, Sweden; 2 Department of Public Health & Clinical Medicine, Occupational and Environmental Medicine, Umeå University, SE-901 85 Umeå, Sweden

**Keywords:** Poisson regression, Occupation, Standardized mortality ratio, Mortality, Cohort study, Mining

## Abstract

**Background:**

A cohort study that examined iron ore mining found negative associations between cumulative working time employed underground and several outcomes, including mortality of cerebrovascular diseases. In this cohort study, and using the same group of miners, we examined whether work in an outdoor environment could explain elevated cerebrovascular disease rates.

**Methods:**

This study was based on a Swedish iron ore mining cohort consisting of 13,000 workers. Poisson regression models were used to generate smoothed estimates of standardized mortality ratios and adjusted rate ratios, both models by cumulative exposure time in outdoor work.

**Results:**

The adjusted rate ratio between employment classified as outdoor work ≥25 years and outdoor work 0–4 years was 1.62 (95 % CI 1.07–2.42). The subgroup underground work ≥15 years deviated most in occurrence of cerebrovascular disease mortality compared with the external reference population: SMR (0.70 (95 % CI 0.56–0.85)).

**Conclusions:**

Employment in outdoor environments was associated with elevated rates of cerebrovascular disease mortality. In contrast, work in tempered underground employment was associated with a protecting effect.

## Background

A cohort study of Swedish iron-ore miners reported that duration of employment time among blue collar workers, especially in underground mining, was associated with lower risks of mortality and incidental cancer [1]. Compared with other workers, mortality from cerebrovascular diseases was lower among long term underground mining blue-collar workers (Rate Ratio: 0.66 (95 % ci 0.49–0.89)). Three explanations for these inverse associations were suggested: a protecting effect from working in a tempered underground environment, a protecting effect from heavy physical workloads, or, a healthy worker survivor effect (HWSE).

The lower risks of cerebrovascular disease associated with duration of underground mining work could be explained by a protecting effect from the more tempered underground climate compared with the outdoor climate. Acute effects of cerebrovascular disease mortality have been explained by cold outdoor temperatures [2, 3]. Workers that remain in longer term employment underground could also be individuals that have better health because of lifestyle factors, beneficial effects from long-term employment or genetic factors. This selection phenomenon of healthier workers remaining in the workforce with a resulting inverse association between cumulative exposure and outcome is called the HWSE [4, 5]. A recent paper from the Kiruna- and Malmberget mines in Sweden showed that adjusting for the HWSE by g-estimation of accelerated failure-time modeling resulted in elevated risks of mortality from respirable dust [6]. Historically, high physical workloads have been associated with mining activities [7]. Several papers have suggested that physically active occupations and lifestyles protect from stroke [8, 9], and colon and rectal cancer [10, 11].

The aim of this study was, to examine if outdoor mining work was associated with elevated rates of cerebrovascular disease mortality. This study will also consider underground work, due to the results from the previous study on this cohort that found a negative association with cerebrovascular disease mortality [1].

## Methods

### The cohort

The Kiruna and Malmberget iron-ore mines are located in the northernmost part of Sweden. They have been in operation since the early 19^th^ century. The mines have similar employment policies and safety routines, and use similar types of equipment. Malmberget has been an underground mine since it began operation. Kiruna was an open-pit mine until the 1950s, when the mining activities successively went underground. Employees were included in the study cohort if they had been employed for at least one year between 1923–1998 at the Kiruna mine, and between 1923–1996 at the Malmberget mine. The cohort has previously been described in detail [12, 13].

Cohort mortality was followed from 1952–2006. Data was collected from the Swedish national cause of death register. The mortality endpoint was cerebrovascular diseases (ICD10: I60–I69), subgroups’ cerebral infarction (ICD10: I63–I64), and cerebral haemorrhage (ICD10: I61). Information on cerebral haemorrhage was only available from 1965. A unique Swedish personal identification number was used to link dates of events and ICD-codes for mortality to each cohort member. Subjects that were not found in the cause of death register or the national population register (*N* = 584) were excluded from the cohort because they had died before 1952 or had emigrated from Sweden. Time at emigration from Sweden after 1952 was censured in the statistical analysis (*N* = 54). Workers for whom information on occupational class (blue-collar worker, white-collar worker, miscellaneous), or work location (inside, outdoor, mining) was not available were excluded (*N* = 130). Work periods were categorized as working underground, working 50 % of the time underground, or other work. We used this work period variable to represent underground work, and the classification procedure has been previously described [12, 13]. Of the remaining workers, 493 of them were employed only in white collar work, and were excluded from the study. The final cohort consisted of 13,000 male workers.

### Classification of work periods into outdoor work

Classification of work location was based on job title, workplace, and year. This information was collected from written and computerized company records. Work location categories were working outdoors, working inside, or working in the mines. Due to the successive change from open to underground mining, all pre-1954 Kiruna mine work periods that were not registered as working inside were classified as working outdoors. This method of classifying work periods into outdoor work implied a possible overlap between the previously defined underground work and this new variable, outdoor work. From 1954 to 1958, blue-collar mining work periods were increasingly classified as working underground with the following weights: 0.1, 0.3, 0.5, 0.7, and 0.9. Classification of work location was performed by three researchers in occupational medicine that were knowledgeable about mine conditions, guided by descriptions of mine history [7].

### Statistical methods

Two log-linear Poisson regression models were calculated. One model was based on cohort data only. The other model also incorporated external rates from the reference population of northern Sweden (male population 1980: 460445).

The model limited to comparisons within the cohort was:

$$ \mathrm{logE}\left({\mathrm{d}}_{\mathrm{k}\mathrm{l}}\right)= \log \left({\mathrm{n}}_{\mathrm{k}\mathrm{l}}\right) + \upalpha +{\mathrm{x}}_{\mathrm{k}}\upbeta + {\mathrm{z}}_{\mathrm{l}}{\upbeta}^{*} $$

where

E(d_kl_): predicted number of outcomes in the cohort per nuisance variable, k, and exposure level variable, l

n_kl_: person years from the cohort per categories, k, and l

α: constant

x_k_β: is a vector of nuisance variables attained age, calendar year, year of first employment, also having been employed as a white collar worker, mine, and cumulative employment time underground and *β* represents the corresponding parameter estimates.

Z_1_β*: is a vector representing cumulative time working outdoors and *β* is the corresponding parameter estimates.

The main predictor (*z*_*l*_β* ) estimated the ratio between categories 5–14, 15–24, and ≥25 years of cumulative employment time outdoor and the reference category 0–4 years. All variables representing cumulative employment time, calendar year, attained age, cumulative employment time underground, and white collar worker (yes, from the first day of employment as a white collar worker and forward, or, no) were analyzed as time-dependent variables. Test of trend was performed by entering a single continuous covariate in this Poisson model, with values representing the mean duration of employment for each category. To evaluate if acute effects of outdoor work could explain elevated rates (deaths occurring close to being employed), a variable coded as 1 (employed) or 0 (not employed) for each time period was included in the model to be able to adjust for possible differences due to deaths that occurred while employed. All deaths within 20 days from the last date of employment were counted as death while employed.

The model that incorporated external rates was:

$$ \mathrm{logE}\left({\mathrm{d}}_{\mathrm{ij}\mathrm{l}}\right)= \log \left({ \exp}_{\mathrm{ij}}\right) + \upalpha +\mathrm{f}\ \left({\mathrm{z}}_{\mathrm{l}}\right) $$

where

E(d_ijl_): predicted number of outcomes in the cohort per year, i, age group, j and category of outdoor work

exp_ij_: expected number of outcomes in the cohort per year, i, age group, j. The expected values were derived from mortality and cancer incidence risks based on the reference population of northern Sweden

α: constant

f(z_l_): a cubic spline function of cumulative time in outdoor work

This model generated smoothed estimates [14] of standardized mortality ratios (SMR) by cumulative employment time categorized into outdoor work. The cubic spline function (f (z_l_)) was based on 3 knots: at 0 years of exposure, at the median number of years of exposure, and at 35 years of exposure. The function was linear from year 35. To complement the adjusted Poisson model described earlier, and to give priority to precision of the estimated SMR, we chose not to include nuisance parameters in this model.

Because of the cohort inclusion criterion of a one year minimum employment time, first year of employment was not included in any statistical analysis as time at risk. The potential for overdispersion was accounted for during Poisson regression estimation of all confidence intervals.

By calculating SMR based on cerebrovascular disease mortality as the outcome, different subgroups of the cohort were compared with the population of northern Sweden. SMR was calculated from cell-specific external rates represented by calendar year in one year classes and age in five year classes (maximum age class, ≥85 years). Because of the wide upper category of ≥85 years in the reference rates, the analyses on subgroups were also performed limited to <85 years of age. 95 % confidence intervals were calculated by assuming that the number of deaths in the cohort followed a Poisson distribution.

Data were analyzed with the statistical software package R (version 3.1.1; R Development Core Team, R foundation for Statistical Computing, Vienna, Austria).

## Results

The study cohort consisted of 13,000 workers representing 443,930 person years. The average age at death of all causes was 67.3 (standard deviation (SD), 14.3) years. The distribution for key variables based on mortality of cerebrovascular diseases as outcome is presented in Table 1.

**Table 1:** Mean (Standard Deviation), age at death and distribution of number of deaths of cerebrovascular diseases

		Cerebrovascular diseases
Mean (SD) age at outcome		72.0 (12.4)
Total number of outcomes		403
Number of outcomes per birth year	−1899	49
	1900–1909	106
	1910–1919	112
	1920–1929	85
	≥1930	51
Number of outcomes per year of first employment	≤1929	112
	1930–1939	60
	1940–1949	62
	1950–1959	137
	≥1960	32
Number of outcomes per cumulative employment time in years	0–4	51
	5–14	75
	15–29	111
	≥30	166
Number of outcomes per mine	Kiruna	246
	Malmberget	157

### The distribution of time classified as outdoor and underground work

**Fig. 1:**
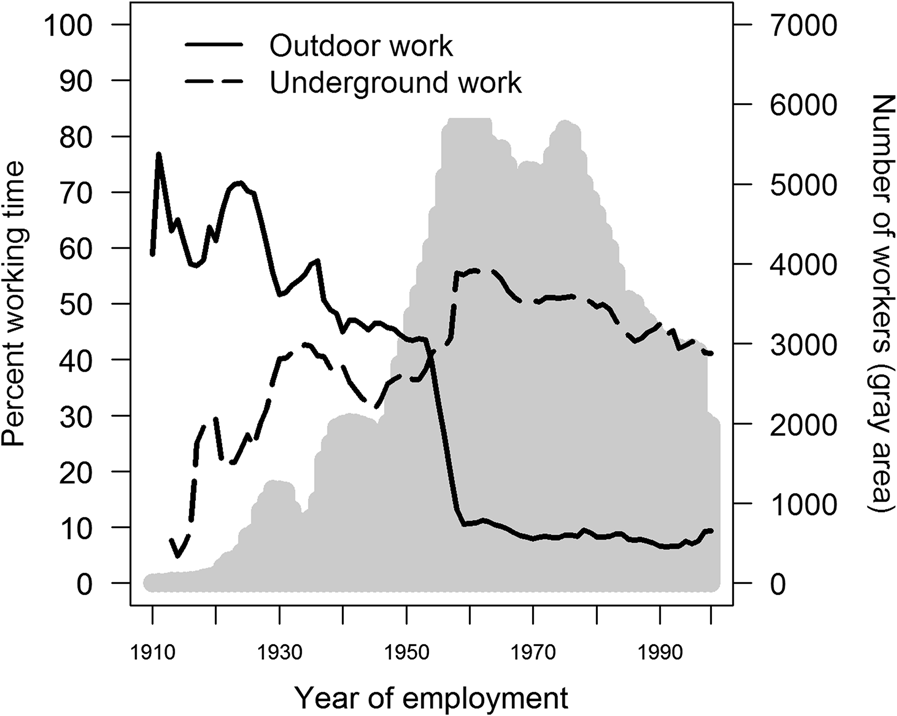
Person year distribution of outdoor and underground work. Gray area represents the number of employed workers

Nineteen percent of the person years, as employed, were classified as “outdoor workers”, compared with 46 % “working inside”, 31 % “mining”, and 3 % “missing”. Working outdoors was most common before 1960 because the Kiruna mine was an open pit mine prior to 1960 (Fig. 1). Of all person time classified as outdoor, 26 % was simultaneously classified as underground work. Of all person years representing employment, 43 % was neither classified as outdoor or underground work. The classification procedures were performed by different experts, and the transition between outdoor and underground work meant that the specific location could be interpreted as outdoor and underground, depending on the criteria for location.

### Outdoor work and the association with cerebrovascular disease mortality

**Fig. 2:**
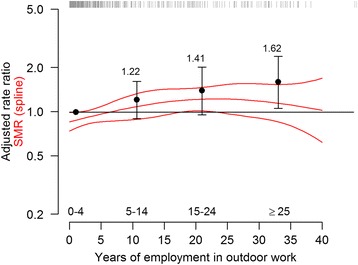
Smoothed estimates of standardized mortality ratios (SMRs) with 95 % confidence intervals (lines) and adjusted point estimates of rate ratios in relation to cumulative time employed outdoors The categorical estimates in the figure are located at the mean employment time (1.0, 10.7, 21.0, 33.1 years). The location of the mortality outcomes in relation to cumulative time employed outdoors is marked on the top of the figure

The adjusted rate ratio estimates for mortality from cerebrovascular diseases increased with cumulative outdoor employment time (Fig. 2 and Table 2). An expanded analysis that included an interaction term between year of first employment and cumulative outdoor employment indicated that rates for workers employed for the first time in later years (1930–1949) more clearly was associated with cumulative outdoor employment compared with earlier years (<1930). This interaction effect between year of first employment and cumulative employment time outdoors did not significantly contribute to the model (p = 0.139, based on a likelihood ratio test for inclusion of the interaction effect). However, the rate ratio estimate for outdoor work ≥25 years compared with 0–4 years was 3.45 (95 % CI 1.55–7.65; 11 deaths: ≥25 years) for workers employed first time 1930–1949. The corresponding estimate was 1.16 (95 % CI 0.73–1.86; 37 deaths: ≥25 years) for workers that were employed first time before 1930. Because of the limited number of outcomes, it was not possible to calculate interaction term-estimates for later years of first employment. We also calculated adjusted rate ratio estimates from Poisson regression by delimiting outcomes to subgroups’ cerebral haemorrhages and cerebral infarctions. Due to the limited number of outcomes, long-term outdoor work was defined as outdoor work ≥15 years. For mortality of cerebral haemorrhages, the adjusted rate ratio for outdoor work 5–14 years (12 deaths) compared to outdoor work 0–4 years (46 deaths) was 1.21 (95 % CI 0.60–2.28) and the corresponding ratio for outdoor work ≥15 years (18 deaths) was 2.52 (95 % CI 1.23–5.04). For mortality of cerebral infarctions, the adjusted rate ratio for outdoor work 5–14 years (23 deaths) compared to outdoor work 0–4 years (71 deaths) was 1.38 (95 % CI 0.83–2.22) and the corresponding ratio for outdoor work ≥15 years (21 deaths) was 1.60 (95 % CI 0.88–2.81).

**Table 2:** Adjusted rate ratio estimates derived from Poisson regression models, with 95 % confidence intervals (CI)

	0–4 years		5–14 years			15–24 years			≥25 years			
	n^a^	Rate ratio	n^a^	Rate ratio	95 % CI	n^a^	Rate ratio	95 % CI	n^a^	Rate ratio	95 % CI	Test of trend, *p*-value
Cerebrovascular diseases	228	1	77	1.22	0.90–1.62	50	1.42	0.97–2.04	48	1.62	1.07–2.42	0.010
Cerebrovascular diseases limited to age ≥ 55	195	1	73	1.26	0.91–1.71	49	1.45	0.97–2.13	47	1.62	1.04–2.47	0.013
Cerebrovascular diseases adjusted for acute effects^b^	228	1	77	1.22	0.91–1.62	50	1.42	0.98–2.04	48	1.63	1.08–2.41	0.008

^a^Number of deaths. ^b^Estimated from Poisson model, included a variable coded 1 if employed and 0 otherwise to control for death occurring while employed

The results for SMR based on splines did not suggest as strong association between outdoor work and cerebrovascular disease mortality as suggested by the adjusted rate ratio estimates (Fig. 2). For short term outdoor work, the rates were lower than the corresponding expected rates derived from the reference population of northern Sweden which contributed to that the SMR, by design, was closer to unit for long term outdoor work compared to the category estimates.

**Fig. 3:**
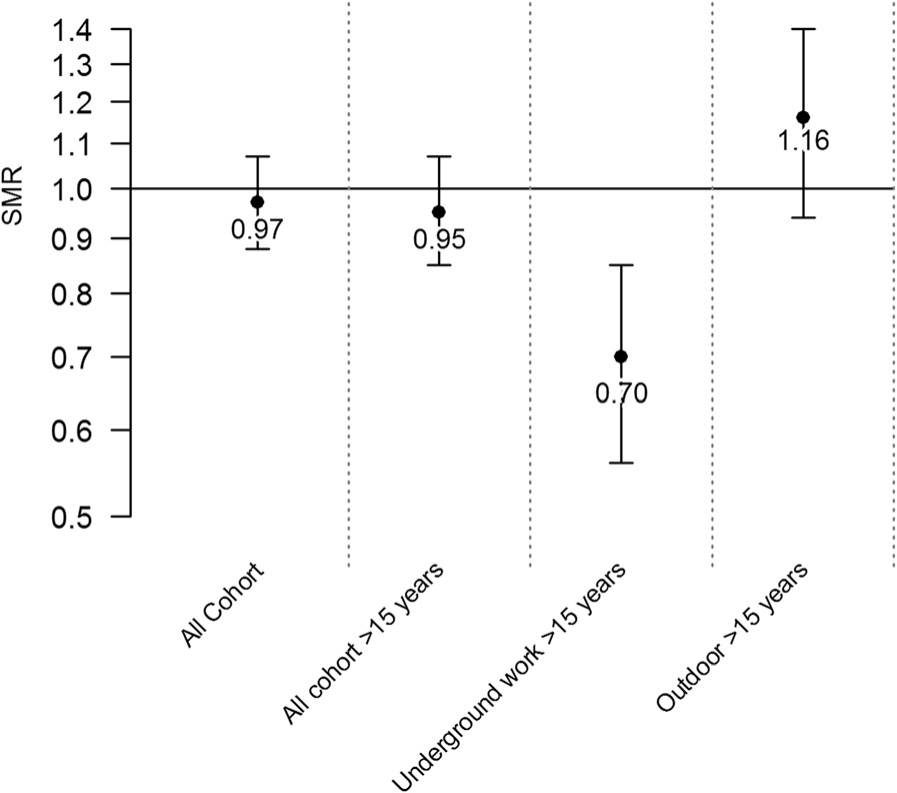
Adjusted rate ratio estimate for cumulative outdoor and underground employment time based on Poisson regression. The estimates are derived from the same model based on the same category levels and the points are located at the mean employment time per category

Figure 3 shows the adjusted rate ratio estimates for cumulative outdoor work together with the corresponding estimates for underground work. The figure clearly illustrates the diverging trends when comparing the estimates of outside and underground work.

Delimiting the adjusted Poisson models to include only age ≥ 55 years did not change the interpretations of the results (Table 2). Adjusting for current employment status in the Poisson model had only small effects on the results (Table 2).

### SMR based on subgroups from the cohort

**Fig. 4:**
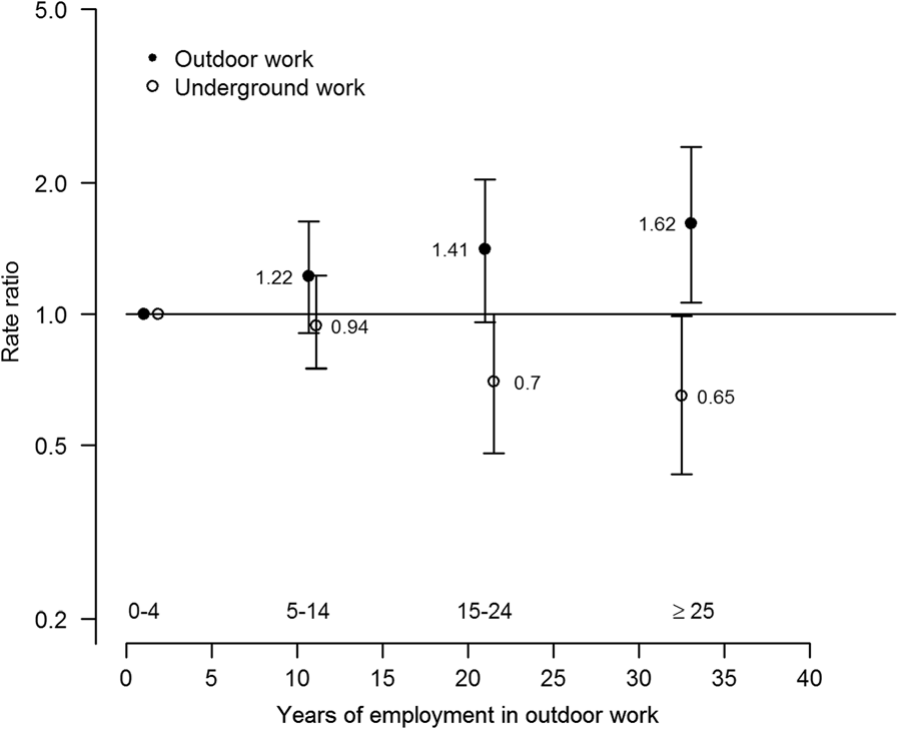
Standardized mortality ratios (SMRs) for cerebrovascular disease based on northern Sweden as reference population. The numbers within the graph are the estimated SMRs

The result for the estimated SMR of mortality from cerebrovascular disease revealed that the rates associated with underground work differed most from the expected rates (Fig. 4). Underground work ≥15 years represented 30 % lower rates: 0.70 (95 % CI 0.56–0.85). Corresponding SMR for outdoor work ≥15 years was 1.16 (95 % CI 0.94–1.40). Delimiting the calculations to include only ages < 85 years yielded similar conclusions, but with slightly lower levels of the estimates overall (data not shown).

## Discussion

The results of this study suggested that there was an association between employments represented by temperature varying outdoor work environments and elevated rates of cerebrovascular disease mortality. The rate of cerebrovascular disease mortality representing cumulative outdoor employment time 25 years or more was 62 % higher (rate ratio: 1.62 (95 % CI 1.07–2.42)) compared with outdoor employment 0 to 4 years (Fig. 2 and Table 2). Moreover, diverging trends between outdoor work and underground work became evident when both variables were included in the same adjusted Poisson model (Fig. 3). There was an indication that the elevated rates for outdoor work were most pronounced for long term outdoor workers employed first time 1930–1949, compared with corresponding workers employed first time before 1930. The adjusted categorical rate ratios for long term outdoor work were noticeably more elevated than the corresponding SMRs based on the reference population (Fig. 2). The rates for short term outdoor work were clearly lower than the reference population of northern Sweden, whereas, the lower limit of the point estimated rate ratio is by design, equal to one. For outdoor work ≥15 years, the rate of cerebral haemorrhages (i.e., the delimiting outcomes for cerebral haemorrhages and cerebral infarctions) was statistically significantly higher than outdoor work 0–4 years – 2.52 (95 % CI 1.23–5.04). The corresponding rate of cerebral infarctions was also elevated, but not statistically significant – 1.60 (95 % CI 0.88–2.81).

The results of the cohort subgroup comparisons of the observed rates and expected rates of cerebrovascular disease mortality derived from the reference population showed that cumulative employment time underground most clearly deviated from the expected rates (Fig. 4). However, the causal link explaining these results could have been connected to both outdoor and underground work. Although adjustments were made for year of employment, attained age, and calendar year in the analysis, outdoor and underground work represented different time-windows of exposure (Fig. 1) as mining methods changed and safety precautions improved. However, the emphasis of the analysis was on earlier years of employment in both groups as the largest proportion of deaths as the result of cerebrovascular diseases occurred among workers who had been employed before 1960 (>90 % in both groups). The relatively smaller deviation in SMR from the reference population for outdoor workers compared with underground workers (Fig. 4) may have been diluted by the 26 % overlap with underground work in the classification procedure. The overlapping work-periods may be explained by the crude classification of outdoor mining, which was defined as all mining in Kiruna before the late 1950s. Moreover, the classification was affected by the fact that some of the mining performed in the earlier periods in Kiruna was drift mining that could be described as a sub-underground mining method.

Cold winter-time temperatures and large seasonal variations in sun light are characteristic of outdoor work in the northernmost part of Sweden. Exposure to cold temperature has been shown to increase blood pressure [15], with several potential mechanisms leading to cerebrovascular disease [16]. However, cold temperatures that result in elevated mortality from cerebrovascular disease act mainly through acute or subacute effects [2]. The monthly distribution of the 304 deaths during one season showed that the three months with the highest frequency of deaths were December, January, and February (31 % of all deaths). Although, adjusting for current status (employed or not employed) did not weaken the association between outdoor work and mortality (Table 2), which implies that outdoor-work employments could have a preserving effect on mortality. We believe that temperature caused hypertension is the risk factor from work that likely best explains these results even if this explanation does require that work has a preserving effect on hypertension.

Smoking increases the risk of cerebrovascular diseases and the associations are strongest in younger ages [9]. The different working conditions and regulations of outdoor and underground work could contribute to different smoking habits by making smoking more or less accessible. We did not have data to adjust for smoking in the analysis. However, a previous study on this cohort showed a SIR of 1.74 (95 % CI 1.52–1.99) for lung cancer among underground workers [1]. The corresponding SIR for outdoor workers was 1.86 (95 % CI 1.53–2.23), a comparable SIR to underground workers. Smoking is a considerably stronger risk factor for lung cancer than it is for cerebrovascular diseases, which indirectly supports that smoking is not a strong confounder in this study. Also, a previous study on workers in the Malmberget mines, which used data on smoking for 2310 workers, found that 75 % of the smokers (1972–1992) had worked underground compared to 71 % of the non-smokers [13], indicating that a possible prohibition on smoking on underground workers did not result in smoking being less common among underground workers. Other known risk factors not possible to adjust for in this study include diabetes, atrial fibrillation, dyslipidemia, asymptomatic carotid stenosis, heredity, and an unhealthy lifestyle factors such as alcohol consumption [9]; these risk factors are harder to causally link with working outdoor or underground (but could still be possible confounders). The hypothesis that excess sunlight could be a cause of mortality from cerebrovascular disease has not been not supported by the literature [17].

A weakness of using cause-specific mortality as a measure has been that the causes of death register contains misclassification errors. In a validation study based on the Swedish cause of death register for 1094 deaths registered in 1995, 0.68 % (95 % CI 0.60–0.77) of all deaths from cerebrovascular underlying causes was correctly classified [18].

### The healthy worker survivor effect

A healthy worker survivor effect (HWSE) [4, 5] could contribute to the reduced mortality rates associated with underground work found in the previous study of this cohort [1]. For cerebrovascular disease, we observed simultaneously elevated rates with outdoor work and reduced rates with underground work, as was found in the previous study. If the HWSE explains this inverse association with underground mining work, it would mirror a shift from underground mining to other categories of employment in the mines that is correlated with increased cerebrovascular disease mortality. The pattern of lower risks for several diseases with longer employment as a blue collar worker, especially underground, that was reported in the previous paper [1], elucidates the need to account for the HWSE when performing studies that focus on cumulative exposures in occupational cohorts.

### Validity of this study

This study was based on iron ore miners from the Kiruna and Malmberget mines and consisted of a relatively large cohort with 443,930 person years and 13,000 workers. The cohort data are of very high quality because of the accuracy of the records of the mining company and because the exposure assessments were performed by occupational health researchers with knowledge about the mines. A study based on workers located in the far reaches of Sweden could be expected to represent a relatively homogeneous population in terms of socio-economic and lifestyle factors, potentially reducing bias from residual confounding. We only included workers who had a valid Swedish unique personal identification number. That is, only Swedish citizens are in the cohort as immigration to Sweden from outside Europe was limited before the 1980s. We did not have any information on the workers’ ethnicity, but a study by Sjölander [19] found that the risk of cardiovascular diseases among the Swedish Sami was slightly lower than the majority population, a finding explained by the active reindeer herding lifestyle. A weakness could have been misclassification of both exposures and for outcomes. As in most epidemiological studies, unmeasured confounding could have influenced the results. Information on hypertension, physical activity, and lifestyle factors other than work would have increased study validity.

## Conclusions

The results of this cohort study revealed that rates of cerebrovascular disease mortality increased with time of employment categorized as outdoor work. This association was also in contrast with the previous observation that work performed in a tempered underground environment has a protecting effect on cerebrovascular disease mortality. However, compared with the northern Sweden reference population, rates for long term outdoor employment were only marginally elevated.

## Abbreviations

HWSE: Healthy worker survivor effect
ICD: International classification of disease
SMR: Standardized mortality ratio
